# Supplementary material for: iMyoblasts for ex vivo and in vivo investigations of human myogenesis and disease modeling
Source: eLife. 2022 Jan 25;11:e70341. doi: 10.7554/eLife.70341 (PMC8789283; doi:10.7554/eLife.70341)
Supplement: Figure 9—source data 2. [file elife-70341-fig9-data2.pptx]

## Slide 1
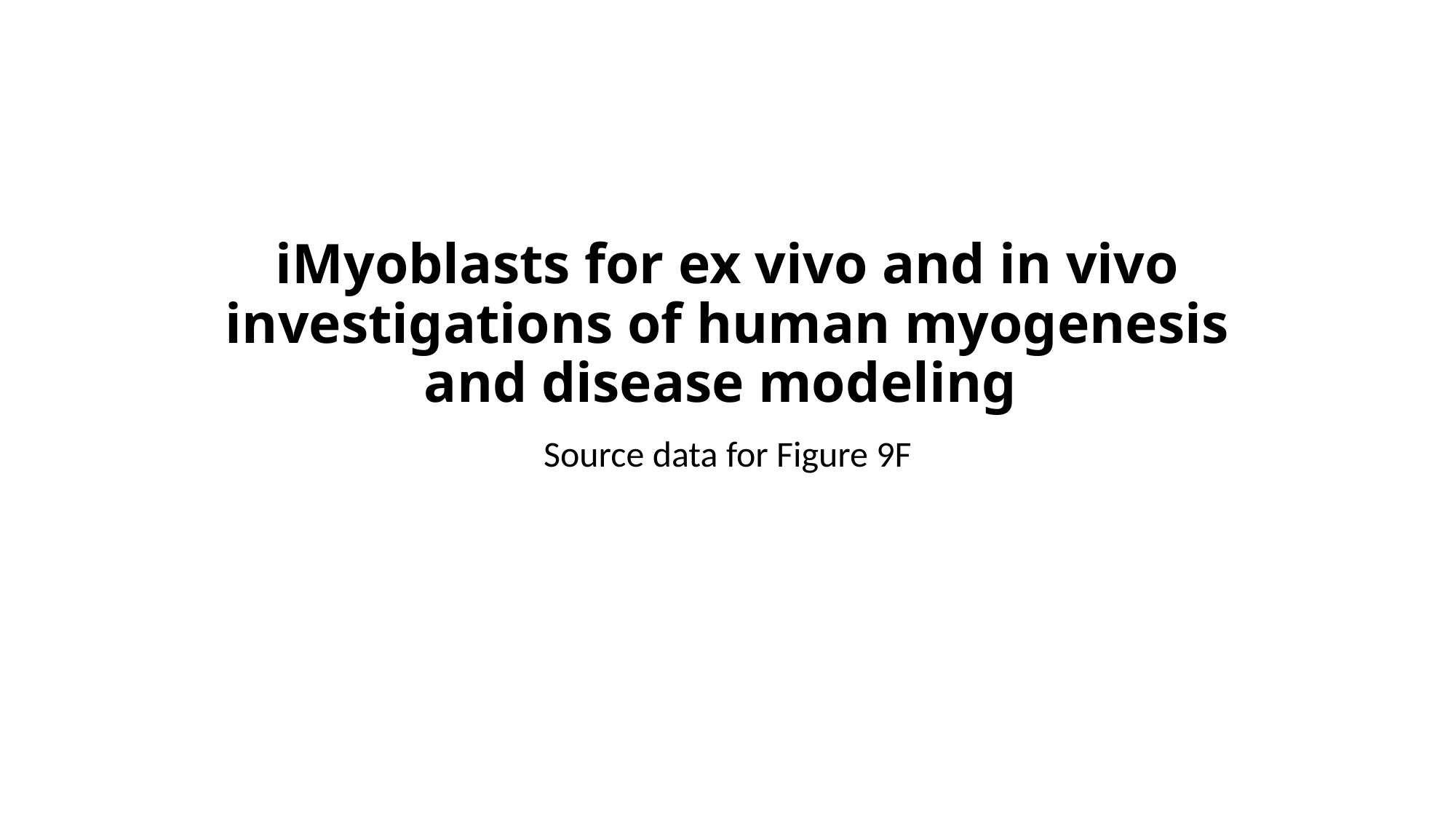

# iMyoblasts for ex vivo and in vivo investigations of human myogenesis and disease modeling
Source data for Figure 9F

## Slide 2
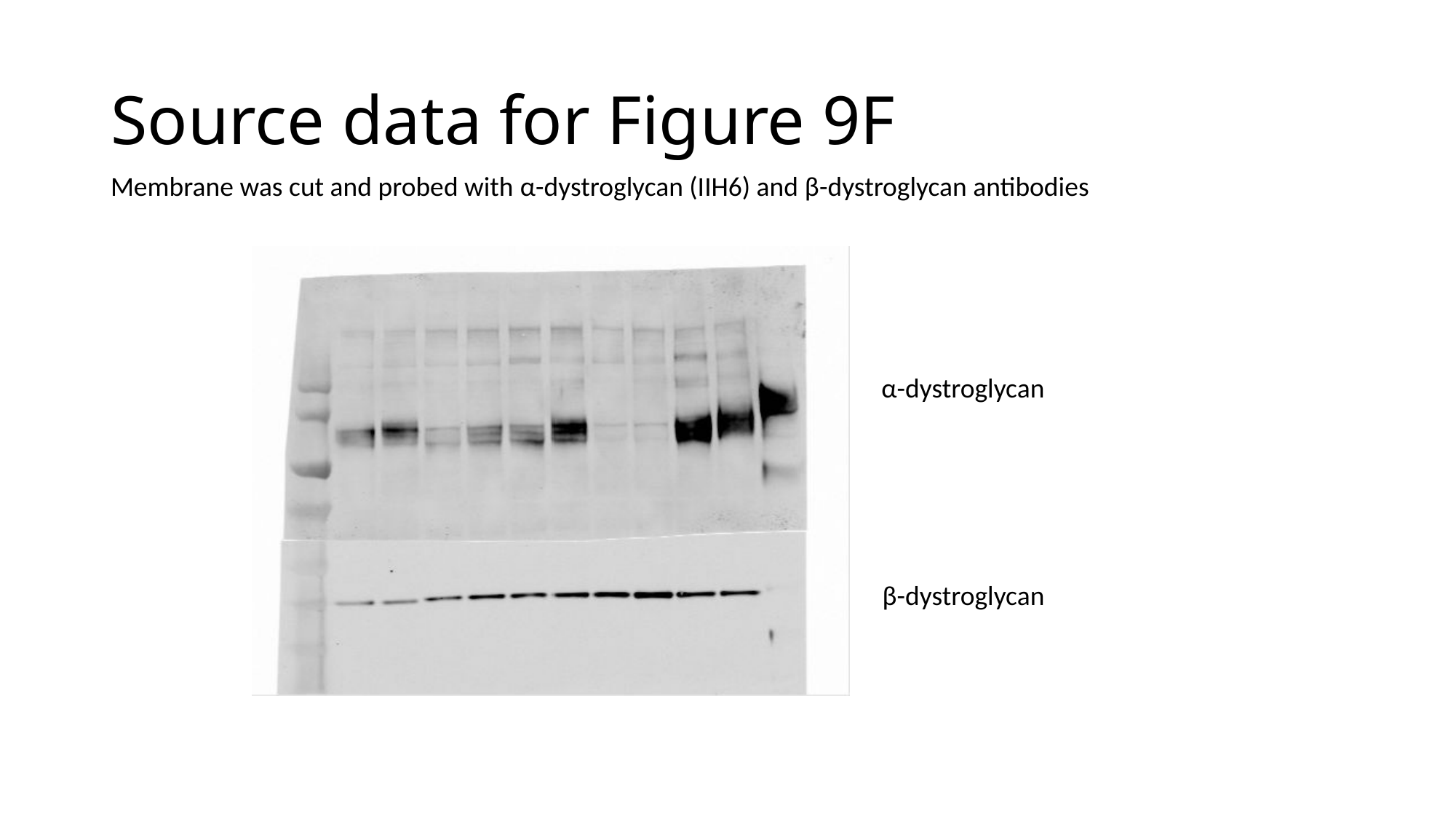

# Source data for Figure 9F
Membrane was cut and probed with α-dystroglycan (IIH6) and β-dystroglycan antibodies
α-dystroglycan
β-dystroglycan

## Slide 3
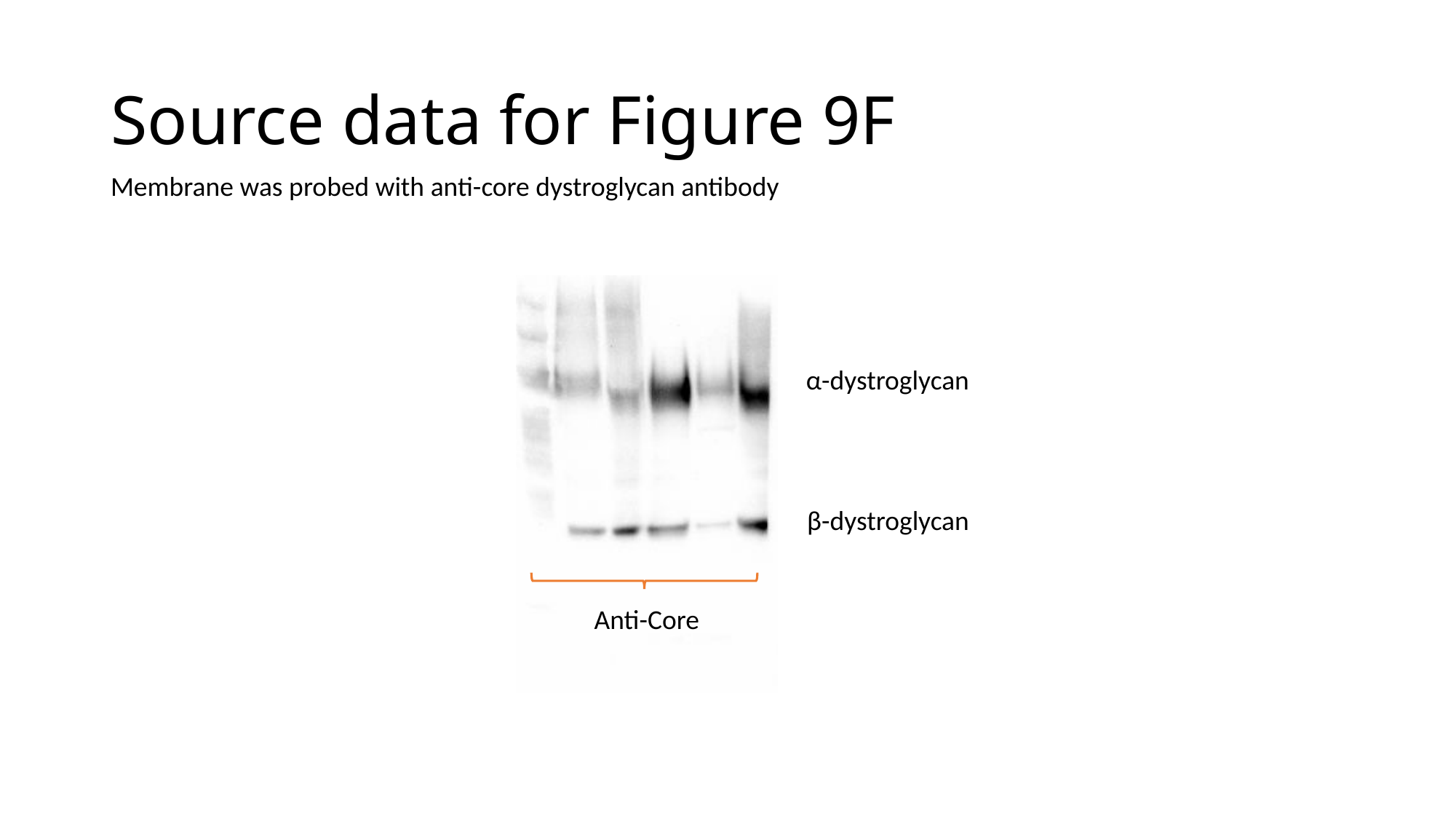

# Source data for Figure 9F
Membrane was probed with anti-core dystroglycan antibody
α-dystroglycan
β-dystroglycan
Anti-Core
